# Chiral Derivatives of Xanthones: Investigation of the Effect of Enantioselectivity on Inhibition of Cyclooxygenases (COX-1 and COX-2) and Binding Interaction with Human Serum Albumin

**DOI:** 10.3390/ph10020050

**Published:** 2017-05-31

**Authors:** Carla Fernandes, Andreia Palmeira, Inês I. Ramos, Carlos Carneiro, Carlos Afonso, Maria Elizabeth Tiritan, Honorina Cidade, Paula C.A.G. Pinto, M. Lúcia M.F.S. Saraiva, Salette Reis, Madalena M.M. Pinto

**Affiliations:** 1 Laboratório de Química Orgânica e Farmacêutica, Departamento de Ciências Químicas, Faculdade de Farmácia, Universidade do Porto, Rua Jorge Viterbo Ferreira nº 228, 4050-313 Porto, Portugal; cfernandes@ff.up.pt (C.F.); andreiapalmeira@gmail.com (A.P.); inesramos89@gmail.com (I.I.R); carlos.mt.carneiro@gmail.com (C.C.); cafonso@ff.up.pt (C.A.); elizabeth.tiritan@iscsn.cespu.pt (M.E.T.); hcidade@ff.up.pt (H.C.); 2 Centro Interdisciplinar de Investigação Marinha e Ambiental (CIIMAR), Universidade do Porto, Edifício do Terminal de Cruzeiros do Porto de Leixões, Av. General Norton de Matos s/n, 4050-208 Matosinhos, Portugal; 3 CESPU, Instituto de Investigação e Formação Avançada em Ciências e Tecnologias da Saúde, Rua Central de Gandra 1317, 4585-116 Gandra PRD, Portugal; 4 REQUIMTE, Departamento de Ciências Químicas, Faculdade de Farmácia, Universidade do Porto, Rua de Jorge Viterbo Ferreira, 228, 4050‑313 Porto, Portugal; ppinto@ff.up.pt (P.C.A.G.P.); lsaraiva@ff.up.pt (M.L.M.F.S.S.); shreis@ff.up.pt (S.R.)

**Keywords:** chiral derivatives of xanthones, cyclooxygenase, albumin, enantioselectivity, docking

## Abstract

Searching of new enantiomerically pure chiral derivatives of xanthones (CDXs) with potential pharmacological properties, particularly those with anti-inflammatory activity, has remained an area of interest of our group. Herein, we describe in silico studies and in vitro inhibitory assays of cyclooxygenases (COX-1 and COX-2) for different enantiomeric pairs of CDXs. The evaluation of the inhibitory activities was performed by using the COX Inhibitor Screening Assay Kit. Docking simulations between the small molecules (CDXs; known ligands and decoys) and the enzyme targets were undertaken with AutoDock Vina embedded in PyRx—Virtual Screening Tool software. All the CDXs evaluated exhibited COX-1 and COX-2 inhibition potential as predicted. Considering that the (*S*)-(−)-enantiomer of the nonsteroidal anti-inflammatory drug ketoprofen preferentially binds to albumin, resulting in lower free plasma concentration than (*R*)-(+)-enantiomer, protein binding affinity for CDXs was also evaluated by spectrofluorimetry as well as in in silico. For some CDXs enantioselectivity was observed.

## 1. Introduction

A key role of chirality in drug design and development is associated with significant effects on the behavior of this kind of compounds in vivo, with enantiomers being able to interact differently with proteins, and other chiral biomolecules [1,2]. These events can be translated into implications in pharmacokinetics (PK) [3], pharmacodynamics (PD) [4] as well as in toxicity [5]. Frequently only one of the two enantiomers exerts the desired effect while the other might be inactive, less potent or even toxic [6,7]. Consequently, enantioselectivity can be considered an essential issue to take into consideration when studying chiral compounds.

There are several classes of compounds illustrating the importance of chirality on both PK and PD events such as non-steroidal anti-inflammatory drugs (NSAIDs) [8]. Considering ketoprofen, for example, the (*S*)-(+)-enantiomer preferentially binds to human serum albumin (HSA), resulting in lower free plasma concentration than (*R*)-(−)-enantiomer [9]. Moreover, the (*S*)-(+)-ketoprofen is several times more potent than the racemate and etodolac, in which the (*S*)-enantiomer has cyclooxygenase (COX) inhibitory activity whereas its antipode does not [10]. Furthermore, some NSAIDs have also demonstrated enantioselectivity in antitumor activity, as for example (*R*)-enantiomer of etodolac binds retinoid X receptor and induces tumor-selective apoptosis in malignant cells [11].

The binding of drugs to plasma proteins is an important parameter since it has implications on drug action in vivo by affecting free concentration in serum [12,13], which has direct implications in pharmacological effects and metabolizing processing [14]. HSA, the most abundant protein in plasma (Mr 66 kDa, concentration 0.53 to 0.75 mM), interacts reversibly with a broad spectrum of drugs, especially neutral and negatively charged hydrophobic compounds [15,16]. According to current point of views in the drug discovery pipeline, the binding of new compounds with HSA at an early stage is of crucial importance, insofar as it affects not only distribution and elimination but also duration and intensity of the pharmacological action of drugs [12,13,17]. Moreover, enantioselectivity for HSA binding has been reported for several drugs such as verapamil and ibuprofen [18], being also predicted by docking studies [19,20]. One group of compounds described with antitumor [21,22,23], anti-inflammatory [24,25,26], among others activities [27,28], concerns xanthone derivatives. Indeed, the xanthone scaffold can be considered a privileged structure [28]. This group of oxygenated heterocyclic compounds can be isolated from natural sources [29,30], including products from marine origin [31], or obtained by synthesis [32,33]. Among them, synthetic chiral derivatives of xanthones (CDXs) have also revealed interesting biological activities [34,35,36,37] and, in some cases, the activity demonstrated to be depending on the stereochemistry of the respective molecules [36,38,39].

**Figure 1:**
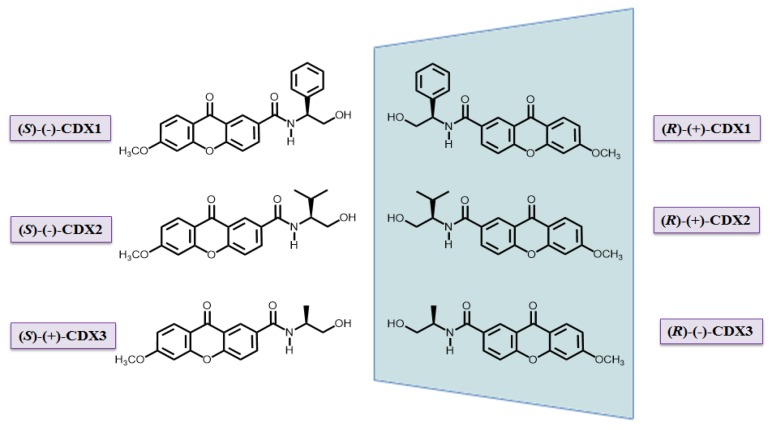
Structures of the enantiomers of CDXs **1**–**3**.

Search of new bioactive CDXs and investigation of enantioselectivity on their biological activity have remained an area of interest of our group [40,41]. Recently, we described the synthesis of new CDXs in enantiomerically pure form, and some of them exhibited growth inhibitory effects on different tumor cell lines as well as enantioselectivity [40]. In this context, herein we described the evaluation of COX inhibition activity and protein binding affinity for three enantiomeric pairs of CDXs (Figure 1).

Molecular modeling studies by docking technique [42,43,44,45] were also carried out in order to understand the interactions of the CDXs with the active site of the referred biological targets and the structural features associated with the chiral recognition.

## 2. Results and Discussion

### 2.1. Cyclooxygenase Inhibition Studies

The effect of CDXs previously obtained in our group [40,41] as inhibitors of membrane located enzymes that might be involved in inflammatory processes, namely COX-1 and COX-2, was evaluated. Three enantiomeric pairs of CDXs were chosen to study the inhibitory effect of both enantiomers of each pair face to biological targets in order to evaluate potency and enantioselectivity. The effect on COXs activity was studied by spectrofluorimetry using a commercial kit measuring the peroxidation activity of COXs. The kit includes isoenzyme-specific inhibitors for distinguishing COX-1 from COX-2 activities.

The results, given as percentage (%) of inhibition and expressed as mean ± standard deviation of two independent experiments, are summarized in Table 1. The overall results indicate that all the CDXs evaluated exhibited COX-1 and COX-2 inhibition potential, although about 20 times less active than indomethacin (positive control). Paired *t*-test was also performed to compare inhibitory effects within each enantiomeric pair to verify the occurrence of enzyme-type and enantioselectivity. Among all the compounds, (*R*)-(+)-CDX2 was the only compound presenting statistically significant enzyme-type selectivity (*p* < 0.05). This enantiomer was more active at inhibiting COX-2 than COX-1. All pairs demonstrated enantioselectivity for COX-1 and |*t*|_calculated_ values were 3.613, 7.249 and 2.891 for CDX1, CDX2 and CDX3 pairs, respectively (*t*_tab(*p* = 0.05; d.f. = 10)_ = 2.228). (*S*)-(−)-CDX1, (*S*)-(−)-CDX2 and (*S*)-(+)-CDX3 were more active than their antipode.

Concerning enantioselectivity for COX-2, the results obtained for the pairs of CDX2 and CDX3 should be highlighted. For instance, the % of COX-2 inhibition for (*S*)-(+)-CDX3 was 93.4 ± 11.4, however the inhibitory effect of (*R*)-(−)-CDX3 was statistically significantly lower. |*t*|_calculated_ value was 2.891 for COX-2 (*t*_tab(*p* = 0.05; d.f. = 10)_ = 2.228). Similarly, (*S*)-(−)-CDX2 was more active than (*R*)-(+)-CDX2 at inhibiting COX-2 (|*t*|_calculated_ = 6.777; *t*_tab(*p* = 0.05; d.f. = 10)_ = 2.228). Accordingly, for these two pairs, weak enantioselectivity was observed. It is important to stress that even though enantioselectivity was observed, its extent was not as evident as that obtained for ketoprofen for instance [10]. 

Molecular docking studies were also performed in order to predict the potential anti-inflammatory activity and to postulate a hypothetical binding model of the tested compounds. The binding affinity between the target and the small molecule was evaluated by the binding free energy approximation (ΔGb, kcal/mol) using AutoDock Vina. The best scored conformation of each compound predicted by AutoDock Vina was selected and further evaluated. The docking score was used to predict the strength of the non-covalent interactions between two molecules after they have been docked (also referred to as binding energy). The docking score is a mathematical approximation of the binding free energy between the ligand and its target.

Diclofenac, indomethacin, naproxen, and piroxicam [46] were used as positive controls and showed negative binding energy values (Table 2). Ligands obtained from the database established more stable complexes with COX-1, with an average binding free energy of −7.8 kcal/mol. Moreover, the docking scores predicted for decoys into the COX-1 was surprisingly low (−7.3 kcal/mol). From the tested compounds, only (*S*)- and (*R*)-CDX3 and (*S*)-CDX2 presented docking scores more negative than the known COX-1 inhibitors indomethacin and piroxicam. However, as only a very small difference was observed between known ligands and decoys scores, this model cannot be used to predict COX-1 inhibition. Hence, more detailed analysis of docking poses and binding mechanisms was performed for the other studied COX isoform: COX-2. Diclofenac, indomethacin, celecoxib, and valdecoxib [46,47] were used as positive controls for COX-2 inhibition. The average binding energy predicted for decoys and known ligands into the COX-2 was −7.6 and −9.3 kcal/mol, respectively (Table 2). Both diclofenac and indomethacin presented −7.9 kcal/mol, whereas celecoxib and valdecoxib exhibited −11.5 and −9.5 kcal/mol, respectively. Among the tested CDXs, (*R*)- and (*S*)-CDX1 exhibited the highest binding affinities and, therefore, lower binding free energies than negative controls.

**Figure 2:**
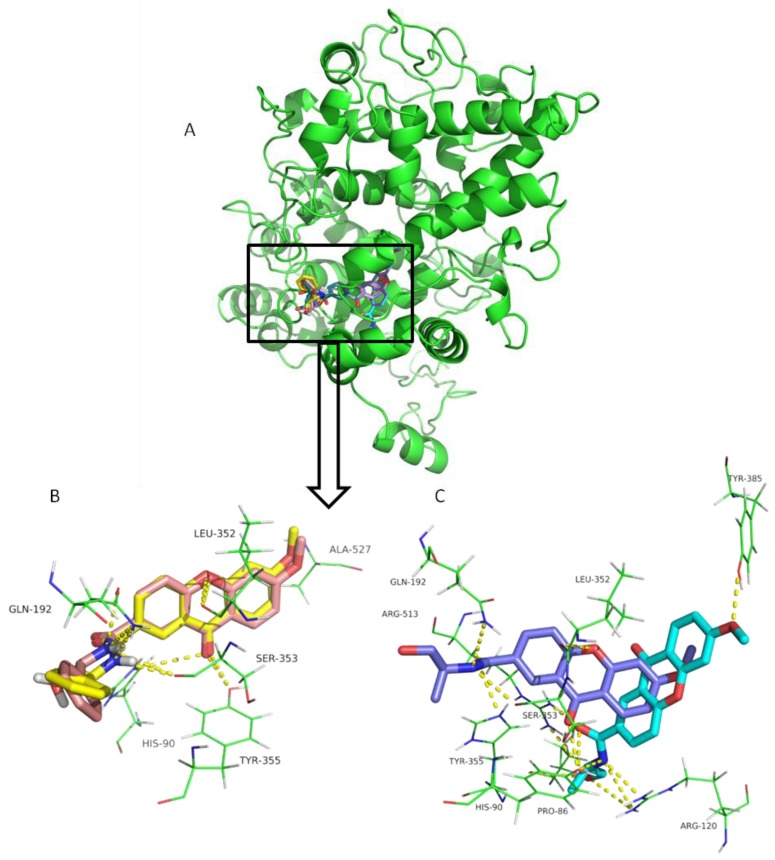
(**A**) COX-2 ribbon representation. (**B**) Comparison of docking poses of CDX1 enantiomers in COX-2 binding site. (*R*) and (*S*) enantiomers are represented in pink and yellow sticks, respectively. (**C**) Comparison of docking poses of CDX3 enantiomers in COX-2 binding site. (*R*) and (*S*) enantiomers are represented in light and dark blue sticks, respectively. Hydrogen interactions are represented as yellow dashes and the residues evolved are represented as green lines and labeled.

Both positive controls and CDXs could dock into the active site of COX-2 successfully (Figure 2A). The binding mode of celecoxib, valdecoxib, indomethacin, and diclofenac (Supplementary Data, Figure S1) are in accordance to the previously reported binding modes. This is of particular importance considering docking accuracy. (*S*)-CDX1 presented the highest binding energy (−8.0 kcal/mol) into the COX-2 model, similar to the known ligands value. Docking energies of −7.8, −7.5, and −7.0 kcal/mol were obtained to the (*R*)-CDX1, (*S*)-CDX3, and (*S*)-CDX2, respectively. CDX3 enantiomers presented very different poses within the binding site of COX-2, whereas CDX1 enantiomers showed a minor difference (Figure 2).

CDX1 enantiomers interact through hydrogen bonds with His90, Leu352, Ser353, Tyr355, and Ala527, also involved in the binding of known anti-inflammatory compounds to COX-2 [48,49,50]. CDX1 enantiomers bind similarly to COX-2 binding pocket, with the xanthone scaffold aligned approximately in the same special position, with a slightly different orientation of the aromatic ring and OH group of the chiral moiety (Figure 2B). (*R*)-CDX1 shows an additional hydrogen interaction between OH and Gln-192, similarly to celecoxib and valdecoxib. On the other hand, CDX3 enantiomers bind in very different poses in COX-2 binding pocket, almost perpendicular to each other (Figure 2C). In fact, (*S*)-CDX3 (Figure 2C, dark blue sticks) binds in a pose similar to CDX1, establishing hydrogen interactions with residues Gln192, His90, Ser353, Leu352, and Tyr355, documented as being important for COX-2 inhibition [50,51,52]. Concerning (*R*)-CDX3, the aromatic backbone projects deep COX active site from the hydrophobic channel, with the C3 chain establishing hydrogen interactions with Arg513, Pro86, and Arg120, and the C6-methoxy group establishing hydrogen interactions with Tyr385 (Figure 2C, light blue sticks), which is important in the catalysis or inactivation of the enzyme [53]. (*S*)-CDX-2 establishes hydrogen interactions with Gln192, Leu352, Ser353, His90, Tyr355, and Arg513; and (*R*)-CDX-2 establishes hydrogen interactions with Tyr385, Tyr355, Arg513, Pro86, and Arg-120 (not shown).

### 2.2. Human Serum Albumin Affinity Studies

HSA-CDX binding parameters are compiled in Table 3. The binding process has reached a completion state as indicated by the *Y_max_* that reached about 100%. For all the compounds, HSA binding occurred spontaneously (ΔG values < 0) in a single binding site (*n* = 1). All compounds presented dissociation constants (K_d_) below 100 µM, which accounts for high affinity binding to HSA [54]. Paired *t*-test was performed to compare K_d_ obtained for (*S*)- and (*R*)-enantiomers of each CDX pair. For all the pairs, there was a statistically significant difference between K_d_ values. Hence, weak enantioselectivity concerning albumin binding was observed. Among all pairs, the highest difference in binding affinity was observed for CDX1 (*ca.* 2.6 fold) as demonstrated by the |*t*|_calculated_ value which was 10.103 (*t*_tab(*p* = 0.05; d.f. = 4)_ = 4.303). The (*S*)-enantiomer presented higher binding affinity compared to the (*R*)-enantiomer. For CDX3 and CDX2, the difference in HSA binding affinity obtained for (*S*)- and (*R*)-enantiomers was less pronounced compared to CDX1. For CDX2, the (*S*)-enantiomer presented slightly higher affinity than the (*R*) one; |*t*|_calculated_ value was 5.484 (*t*_tab(*p* = 0.05; d.f. = 4)_ = 2.776). On the contrary, (*R*)-(−)-CDX3 has shown slightly higher affinity than (*S*)-(+)-CDX3. In this case, |*t*|_calculated_ value was 3.713 (*t*_tab(*p* = 0.05; d.f. = 4)_ = 2.776). 

**Figure 3:**
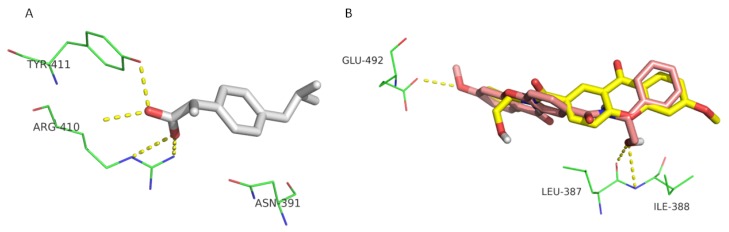
Interactions of crystallographic (*S*)-ibuprofen (yellow sticks) (**A**) and docked CDX1 enantiomers (**B**) with HSA. (*R*)- and (*S*)-enantiomers are represented in pink and yellow sticks, respectively. Hydrogen interactions are represented as yellow dashes and the residues evolved are represented as green lines and labeled.

Regarding computational studies, drugs that are described as having high affinity to HSA lead to docking scores between −4.4 kcal/mol (Iophenoxid acid) and −8.5 kcal/mol (warfarin) (Table 3). CDXs presented scores from −7.0 to −7.3 kcal/mol, and therefore it is hypothesized that they will have high affinity to albumin target. There is an offset between the ∆G binding and the docking scores. This relates to the ability of the docking algorithm to predict the strength of ligand binding to the protein target, and therefore, other scoring functions will be used in the future to increase the accuracy.

(*S*)-Ibuprofen binds albumin through hydrogen interactions with Arg-140, Tyr-411, and Lys-414 (Figure 3A), residues described as being involved in binding of substrates to HSA [55]. The present study indicates that CDXs fit within the hydrophobic pocket of subdomain IIIA, presenting low negative docking scores. This groove was selected for the docking studies as it was described as being the binding pocket for (*S*)-ibuprofen and most ligands [56]. CDXs bind in a similar position in the binding groove, with the central xanthone ring aligned with ibuprofen ring. The binding of CDX enantiomers in HSA subdomain IIIA present differences concerning the number of hydrogen interactions. For example, the complex (*R*)-CDX1-HSA is stabilized by three hydrogen-bond interactions with residues Leu-430, Ser-489, and Asn-391, already described as being involved in the binding of drugs to HSA [57,58,59], whereas (S)-CDX1 lacks those interactions (Figure 3B). Therefore, there is concordance between in silico and in vitro studies, as enantiosselectivity can be found on the binding of CDX1 to HSA.

## 3. Materials and Methods

### 3.1. Compounds

CDXs (Figure 1) were synthesized in enantiomerically pure form [40,41]. Briefly, a carboxyxanthone derivative, with the structure based on some bioactive xanthones from marine origin [31] was coupled with both enantiomers of commercially available chiral building blocks using *O*-(benzotriazol-1-yl)-*N-N-N’-N’*-tetramethyluronium tetrafluoroborate as coupling reagent and a catalytic amount of triethylamine in tetrahydrofuran, at room temperature. Liquid chromatography using different types of chiral stationary phases was used to determine the enantiomeric purity of the synthesized compounds [60,61], achieving enantiomeric excess values higher than 99%.

### 3.2. In vitro Cyclooxygenase Inhibition Studies

The evaluation of the inhibition of COX-1 and COX-2 by CDXs was conducted using the commercially available COX (ovine/human) Inhibitor Screening Assay Kit (Cayman Chemical, Michigan, MI, USA). Briefly, the assay implies an enzymatic immunoassay based on the competition between prostaglandins (PGs) and a PG-acetylcholinesterase (AChE) conjugate (PG tracer), which is then evaluated by the addition of Ellman’s reagent. All the solutions required for the experiment were prepared according to the manufacturer’s instructions. Each compound was analysed in two independent days, in triplicate. CDX working solutions were prepared in DMSO to a final concentration of 20 µM. Indomethacin (1 µM) was used as positive control. Absorbance measurements at 412 nm were performed in a Synergy HT microplate reader (Bio-Tek Instruments, Winooski, VT, USA) operated with Gen5 software (Bio-Tek Instruments).

### 3.3. Interaction with Human Serum Albumin by Fluorescence Quenching

Evaluation of the binding of CDXs to HSA was based on the quenching of HSA intrinsic fluorescence. Phosphate buffer consisting of 7.5 mM Na_2_HPO_4_, 1.5 mM KH_2_PO_4_ and 140 mM NaCl (pH 7.4) was used in the preparation of CDX solutions. Solutions of HSA fraction V (Sigma-Aldrich, St. Louis, MO, USA) were prepared in water. Briefly, 500 µL of HSA (fixed final concentration 2 µM), increasing volumes of drug solution (*n* = 13, 0–200 µM) and phosphate buffer solution (pH 7.4) were mixed to a final volume of 1500 µL. The corresponding blank solutions were identically prepared and analysed in the absence of the drug. Fluorescence emission spectra were recorded in the range of 300–450 nm upon excitation at 295 nm. Excitation spectra were also recorded between 220 and 310 nm with emission set at 330 nm. For individual measurements, excitation and emission wavelength were set at 295 nm and 330 nm, respectively. For each measurement, fluorescence emission was automatically acquired during 180 s (5 nm bandwidth). For all compounds, UV-vis absorption spectra (200–500 nm) were recorded. These measurements were used to correct the fluorescence intensity values due to inner filter effects at the excitation wavelength [62]. All experiments were performed at room temperature (25 ± 1 °C). Fluorescence and absorbance measurement were performed in a LS-50B spectrometer (Perkin Elmer, Waltham, MA, USA) and a V-660 spectrophotometer (Jasco, Easton, MD, USA) respectively.

#### Assessment of HSA-CDX Binding Parameters

HSA-CDX binding parameters were calculated using the Origin 8.5.1 software (8.5.1, Northampton, MA, USA). The fitting of the experimental values was made according to the Langmuir binding equation (Equation (1)) to calculate HSA binding parameters:

(1) $$\left[\mathrm{HSA}-\mathrm{CDX}\right]=\frac{\left[\mathrm{HSA}\right]}{1+\;\frac{K_{d}}{\left[\mathrm{CDX}\right]}}$$

where [HSA], [CDX] and [HSA-CDX] are given in μM and K_d_ corresponds to the dissociation constant of HSA-CDX complexes.

In terms of fluorescence quenching mediated by the compound, the previous Langmuir isotherm can be rewritten as follows (Equation (2)):

(2) $$\%\mathrm{quenching}=\frac{{y_{\max}}^{n}}{1+\;\frac{K_{d}}{\left[\mathrm{CDX}\right]}}$$

where *y*_max_ corresponds to the highest percentage of quenching induced by a given compound and *n* accounts for the number of biding sites of the enzyme to the CDX. Finally, free Gibbs energy (ΔG) was also determined for all the interactions.

### 3.4. Computational Studies

#### 3.4.1. Preparation of CDXs, Controls, Decoys, and Macromolecules

The six CDXs and several known inhibitors (Table 1 and Table 2) were drawn and minimized using Universal Force Field (UFF) of Rappé and coworkers [63] which consists of a molecular mechanics (MM) force field that includes parameterization for the entire periodic table. The calculation is finished when the gradient between any two successive steps in the geometry search is less than 10^−1^ kcal/mol/Å or the maximum steps are reached, whichever comes first. The line search used is the Broyden-Fletcher-Golfarb-Shanno search which uses an approximate Hessian matrix to guide the search. Charges were calculated using gasteiger method [64] available in Chimera [65].

COX-1 and COX-2 decoy and ligand sets were downloaded from *A Directory of Useful Decoys (DUD)* [66], a database from the University of California, San Francisco. A hundred decoys and a hundred ligands were used for docking simulations with COX-2. Fifty decoys and the twenty three available ligands were used for docking simulations with COX-1. These molecules were used with no further manipulation.

The X-ray crystal structures of COX-1 (PDB code: 3n8x) and COX-2 (PDB code: 1cx2) were downloaded from the Protein Data Bank of Brookhaven [67]. An additional docking study was performed using HSA as target (PDB code: 2bxg), and azaprozone, diazepam, fusidic acid, ibuprofen, iophenoxid acid, naproxen, and warfarin as positive controls.

#### 3.4.2. Docking

Docking simulations between the CDXs, the anti-inflammatory targets, and HSA were undertaken in AutoDock Vina (Molecular Graphics Lab, La Jolla, CA, USA) [68]. AutoDock Vina considered the target conformation (biomacromolecule) as a rigid unit while the ligands were allowed to be flexible and adaptable to the target. Vina searched for the lowest binding affinity conformations and returned nine different conformations for each CDX. The lowest binding energy docking poses of each compound were chosen. AutoDock Vina was run using an exhaustiveness of 8 and a grid box with the dimensions of X: 21.6, Y: 22.3, Z: 20.2 for COX-1; X: 22.9, Y: 23.7, Z: 21.7 for COX-2; and X: 18.0, Y: 19.0, Z: 19.0 for HSA. PyMol v1.3 (Schrödinger, New York, NY, USA) [69] and Chimera (UCSF, San Francisco, CA, USA) [65] were used for visual inspection of results and graphical representations.

## 4. Conclusions

All CDXs evaluated exhibited COX-1 and COX-2 inhibition in in vitro assays. Generally, the inhibitory effects were very similar for both COXs, with exception of CDX2 enantiomeric pair. Among all the compounds, (*R*)-(+)-CDX2 was more active at inhibiting COX-2 than COX-1. Interestingly, all pairs demonstrated enantioselectivity for COX-1. Concerning COX-2, the percentage of inhibition for (*S*)-(−)-CDX2 and (*S*)-(+)-CDX3 was higher. Accordingly, for CDX2 and CDX3 pairs enantioselectivity for COX-2 was also observed.

Regarding in in silico studies, no significant difference was found between known ligands and decoys docking scores on COX-1, therefore, no reliable conclusions can be taken from test ligands binding affinity to COX-1. However, regarding docking studies with COX-2, CDX-3 enantiomers as they presented very different poses within the binding site of the enzyme, it is reasonable to predict enantioselectivity been in accordance to in vitro studies.

Additionally, all CDXs demonstrated to bind with high affinity to HSA in in vitro assays and weak enantioselectivity was observed for all enantiomeric pairs. This effect was particularly evident for CDX-1 pair. The in silico studies also confirmed that CDXs bind to HSA, as they have docking scores similar to positive controls such as ibuprofen and diazepam; and CDX1 enantiomeric pair exhibited enantioselectivity. Regarding HSA affinity studies, agreement between in silico and in vitro data (activity and enantioselectivity) was achieved. Moreover, useful information about the mechanism of molecular recognition for both COX and HSA was obtained. Even though statistical analysis demonstrated only a trend for enantioselectivity, we envision that the introduction of molecular modifications supported by docking studies might enhance those differences.

Taking into account the results of this study, it can be concluded that new knowledge was added in the field of CDXs as potential anti-inflammatory agents, paving a very interesting way to understand the enantioselectivity of this family of compounds facing to COX and HSA.

## Figures and Tables

**Table 1:** Inhibitory effects of CDXs on COX-1 and COX-2.

CDX	COX-1	COX-2
(S)-(−)-CDX1	87.6 ± 2.1	80.1 ± 12.8
(R)-(+)-CDX1	79.6 ± 5.0	84.7 ± 5.7
(S)-(−)-CDX2	82.9 ± 5.2	85.7 ± 4.5
(R)-(+)-CDX2	66.8 ± 1.6	73.2 ± 0.4
(S)-(+)-CDX3	91.7 ± 10.7	93.4 ± 11.4
(R)-(−)-CDX3	75.2 ± 9.0	75.1 ± 7.2
Indomethacin	83.2 ± 6.4	80.7 ± 9.5

Values correspond to percentage of enzyme inhibition (mean ± standard deviation). Each compound was analyzed in triplicate in two independent days. The concentration of CDXs was 20 µmol/L. Indomethacin 1 µmol/L was used as positive control.

**Table 2:** Docking scores of corresponding test compounds at COX-1 and COX-2 targets.

Compounds		Docking Score (kcal/mol)	
		COX-1	COX-2
Known ligands	Diclofenac	−6.1	−7.9
	Indomethacin	−5.1	−7.9
	Naproxen	−7.8	
	Piroxicam	−5.2	
	Celecoxib		−11.5
	Valdecoxib		−9.5
Ligands from database		−7.8	−9.3
Decoys from database		−7.3	−7.6
(*R*)-(+)-CDX1		−4.2	−7.8
(*S*)-(−)-CDX1		−4.5	−8.0
(*R*)-(+)-CDX2		−3.4	−6.5
(*S*)-(−)-CDX2		−5.4	−7.0
(*R*)-(−)-CDX3		−5.3	−6.9
(*S*)-(+)-CDX3		−5.6	−7.5

**Table 3:** HSA binding parameters obtained for the CDXs and predicted docking scores between HSA and CDXs.

Compound		K_d_	*Y*_max_	∆G Binding	Docking Score (kcal/mol)
Known ligands	Azaprozone				−5.9
	Diazepam				−7.1
	Fusidic acid				−5.8
	(*S*)-Ibuprofen				−7.3
	Iophenoxid acid				−4.4
	Naproxen				−7.9
	Warfarin				−8.5
(*R*)-(+)-CDX1		61.8 ± 6.5	109.6 ± 1.6	−2.4 ± 0.2	−7.3
(*S*)-(−)-CDX1		23.6 ± 0.8	105.3 ± 0.4	−1.9 ± 0.1	−7.0
(*R*)-(+)-CDX2		29.2 ± 0.9	108.2 ± 0.2	−2.0 ± 0.1	−7.2
(*S*)-(−)-CDX2		24.7 ± 1.1	107.4 ± 5.4	−1.9 ± 0.1	−7.2
(*R*)-(−)-CDX3		26.4 ± 1.2	113.2 ± 1.4	−1.9 ± 0.1	−7.2
(*S*)-(+)-CDX3		31.4 ± 2.0	116.2 ± 0.6	−2.0 ± 0.2	−7.0

Values correspond to the mean ± standard deviation of triplicate runs; K_d_ corresponds to the dissociation constant of CDX-HSA in μM; *Y*_max_ corresponds to the maximum percentage of HSA fluorescence quenching; ∆G for binding expressed in kcal/mol.

## Supplementary Materials

The following are available online at http://www.mdpi.com/1424-8247/10/2/50/s1.
