# Supplementary material for: Chiral Derivatives of Xanthones: Investigation of the Effect of Enantioselectivity on Inhibition of Cyclooxygenases (COX-1 and COX-2) and Binding Interaction with Human Serum Albumin
Source: Pharmaceuticals (Basel). 2017 May 31;10(2):50. doi: 10.3390/ph10020050 (PMC5490407; doi:10.3390/ph10020050)
Supplement: Supplementary file 1 [file pharmaceuticals-10-00050-s001.docx]

**Supplementary Materials: Chiral Derivatives of Xanthones: Investigation of the Effect of Enantioselectivity on Inhibition of Cyclooxygenases (COX-1 and COX-2) and Binding Interaction with Human Serum Albumin**

Carla Fernandes ^1,2^, Andreia Palmeira ^1,2^, Inês I. Ramos ^1^, Carlos Carneiro ^1^, Carlos Afonso ^1,2^,
Maria Elizabeth Tiritan ^1,2,3^, Honorina Cidade ^1,2^, Paula C.A.G. Pinto ^4^, M. Lúcia M.F.S. Saraiva ^4^, Salette Reis ^4^ and Madalena M.M. Pinto ^1,2,^*

| 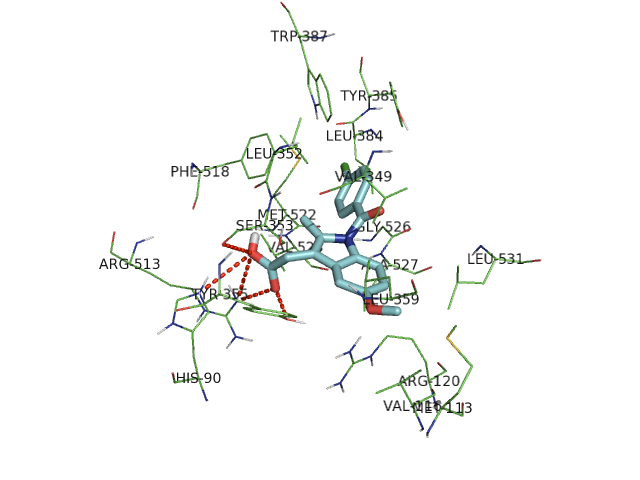  (**a**) | **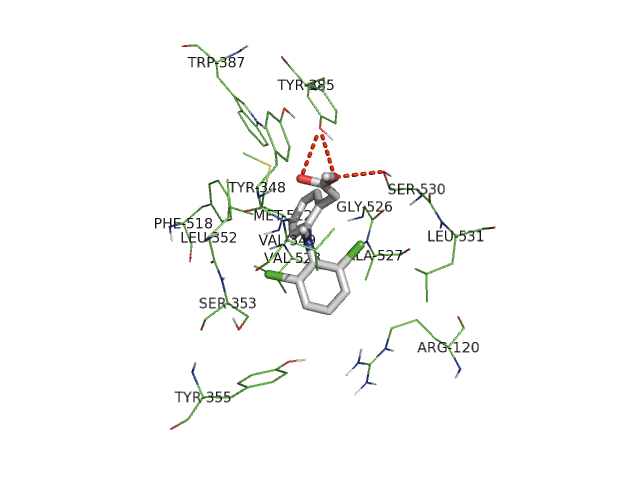**  (**b**) |
| --- | --- |
| **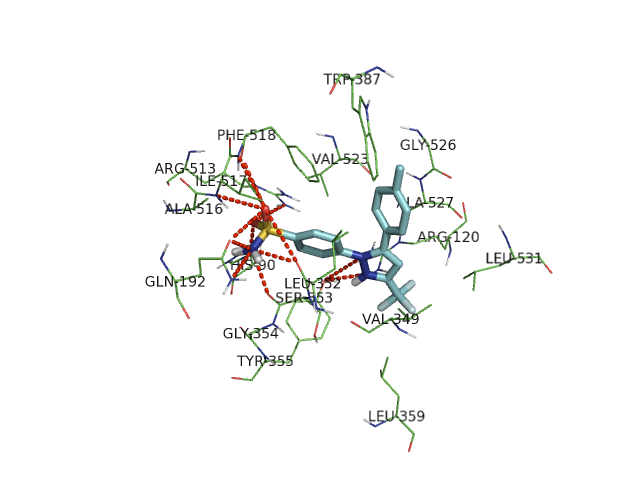**  (**c**) | **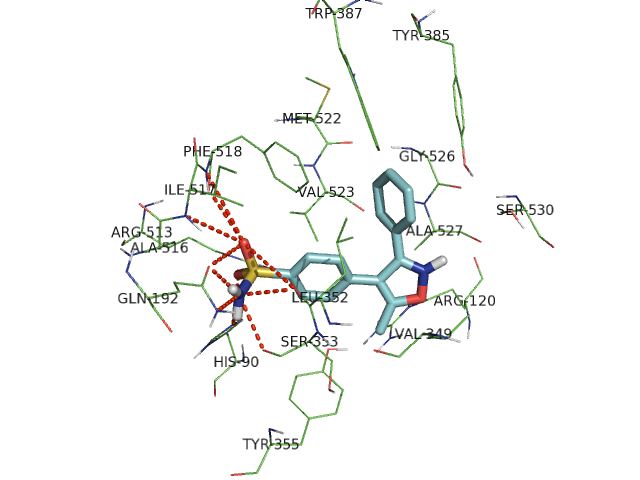**  (**d**) |

**Figure S1.** Hydrogen bonding interactions between COX-2 and (**a**) indomethacin, (**b**) diclofenac, (**c**) celecoxib and (**d**) valdecoxib.
